# Management of an Unusual Metastasis of Cervical Cancer in the Adrenal Bed With Stereotactic Ablative Body Radiation Therapy

**DOI:** 10.7759/cureus.22178

**Published:** 2022-02-13

**Authors:** Raul Puente-Vallejo, Pamela Ochoa, Cristina Núñez, Luis De Los Reyes

**Affiliations:** 1 Department of Cancer Research, Faculty of Health and Life Sciences, Universidad Internacional del Ecuador, Quito, ECU; 2 Department of Radiation Oncology, Hospital Solón Espinosa Ayala (SOLCA), Quito, ECU; 3 Department of Medical Research, NeurALL Research Group, Quito, ECU

**Keywords:** vmat, ablative therapy, adrenal bed metastases, sabrt, cervical cancer

## Abstract

Uterine cervical carcinoma is an important type of cancer among Ecuadorian women, especially in adult women. Survival rates have improved with the development of radiotherapy, surgical techniques, and chemotherapy. However, recurrence and/or metastasis are not unusual phenomena. Frequent sites of metastasis are the lungs, regional lymph nodes, and bones. Atypical locations can also occur on solid organs, such as adrenal glands. Treatment for the rare complication that is adrenal metastasis is individualized, it can include surgical resection, chemotherapy, local ablation, or different types of radiotherapy.

We aimed to report a case of an Ecuadorian woman from Quito city with a diagnosis of cervical carcinoma diagnosed in 2009, treated surgically and with adjuvant chemotherapy. Her progression was monitored with medical controls with no recurrence until 2018, when she relapsed with a metastatic invasion of the pelvic ganglia and the surroundings of the abdominal aorta, with a histopathologic diagnosis of adenocarcinoma. She was then treated with chemotherapy and radiotherapy until June 2019. In 2020, she went through a splenectomy and left adrenalectomy to treat vascular thrombosis. In 2021, 37 x 15 mm mass was discovered in the surgical bed of the previously removed adrenal gland. It was treated as an oligometastatic carcinoma with stereotactic body radiotherapy (SBRT) by a linear accelerator.

## Introduction

Cervical carcinoma is the third most frequent and deadly carcinoma among Ecuadorian women. The incidence is elevated in Ecuador compared with the rest of the world, being at a rate of 18.5 cases per 100,000 inhabitants [1,2]. There were 449 deaths by cervical carcinoma registered in Ecuador in 2015, with an average patient age of 60.3 years [2]. Metastases develop in 15-61% of patients, usually within the first two years after the end of the treatment. This complication can be treated as an oligometastatic disease or as a disseminated metastasis [3].

Chemotherapy in combination with an angiogenesis inhibitor (bevacizumab) is recommended for women with recurrent cervical carcinoma or advanced metastases, with a significant improvement in global survival [3]. In cases that can be classified as oligometastatic, the rescue treatment could include surgery, stereotactic body radiotherapy (SBRT), or brachytherapy, depending on the lesion localization and the clinical conditions of the patient [4,5].

Although there are no clinical reports in the literature about the treatment of adrenal bed metastases in patients with adrenalectomies, due to the presumable dissemination route and the site of metastases, this case can be approached as if it was a metastasis to the adrenal gland. Considering metastasis to the adrenal gland, many studies have confirmed an increase in the overall survival (OS) rate after the adrenalectomy in patients with no dissemination to other organs. The benefit of this surgery is however disputed, the outcomes depend on the kind of surgical technique used and the primary tumor site. The identification of patients that could benefit from a surgical resection is still difficult, but the survival rate difference between open surgery and laparoscopy is not significant [6-8].

SBRT allows delivery of high doses of radiation and the ablation of the tumor at a time. The use of this type of radiotherapy is well-established in the treatment of oligometastatic carcinomas of the lungs and liver, but the evidence of its use in adrenal glands is poor [4,5,9-11]. In the first metanalysis that collects the outcomes of SBRT use for adrenal metastases, it was found that the treatment is associated with an excellent one-year local control (LC) of the carcinoma [12]. Additionally, it helped with the palliative treatment of the pain and the reduction of the mass volume [13]. The clinically significant toxicity rate was 1.8%. There were no reports of severe renal or adrenal toxicity. SBRT provides high local control and an acceptable toxicity rate, which is auto-limited in the acute toxicity phase [12].

Many studies have explored the effect of biologically effective dose (BED) on the local control of the mass. It has been observed that the highest BED values are associated with better control of the tumor size. The dose used for pelvic and abdominal masses that presumably includes adrenal metastases was 30-60 Gy in three to 10 fractions [14-18]. 

We now report a case of a patient from Quito, Ecuador, with cervical cancer. She was first treated in 2009, and she had a relapse with metastases in the retroperitoneal ganglia in 2018, but it was well controlled. That same year, she had a metastatic invasion of the left adrenal bed, however, she was not a candidate for surgical resection, as she has had a splenectomy and left adrenalectomy because of a history of thrombosis. The mass was then managed with SBRT, administered by a linear accelerator (LINAC). This treatment was decided based on the recommendations for the management of adrenal gland metastases, as there are no recommendations for the specific condition of our patient [16,17]. Clinical target volume (CTV) and internal target volume (ITV) modifications had to be done because of the condition of loss of the natural barrier that the absence of the left adrenal gland and the surgical manipulation of the site implied. The resolution of this case was handled in spite of the limitations generated by the coronavirus disease 2019 (COVID-19) pandemic. According to the imaging studies, the mass response was favorable to the treatment and the patient was clinically and functionally better.

## Case presentation

We report the case of a 77-year-old patient who was born in Quito, Ecuador, and resides currently there. She presented a pathological history of arthrosis of the left knee, managed with corticoids and non-steroidal anti-inflammatory drugs (NSAIDs), on stand-by for a surgical resolution. She also had a history of uterine cervix carcinoma diagnosed in 2009, currently presenting metastases. She does not refer to intoxication history, no family history of carcinomas, or other pathologies of importance.

In 2009, uterine cervical carcinoma was initially managed by surgery and an adjuvant chemotherapy regime. She had medical appointments to monitor the recovery progress with no abnormalities until 2018 when she relapsed. She presented then pelvic adenopathies and on the surroundings of the abdominal aorta, with a histopathological report of metastatic adenocarcinomas. Those metastases were treated with platinum-based chemotherapy and 50 Gy radiotherapy fractioned in 25 pelvic and retroperitoneum sessions until June 2019, with a favorable response. She underwent splenectomy and left adrenalectomy to treat a splenic and superior adrenal arteries thrombosis in March 2020.

**Figure 1:**
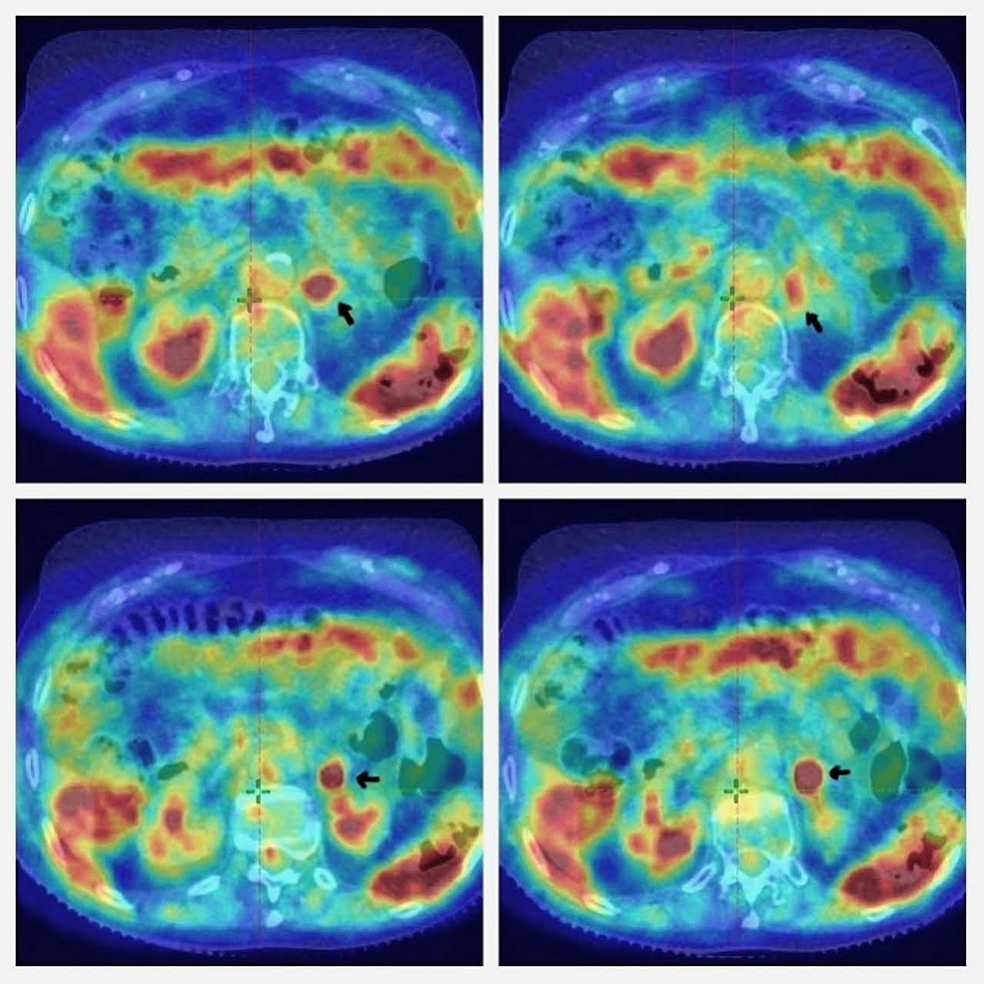
Pre-treatment abdominal PET-CT Apart from the physiological uptakes, a region of high uptake can be identified in the left adrenal bed (arrow). No other findings that may suggest pathology are identified in these images. PET-CT: positron emission tomography-computed tomography

**Figure 2:**
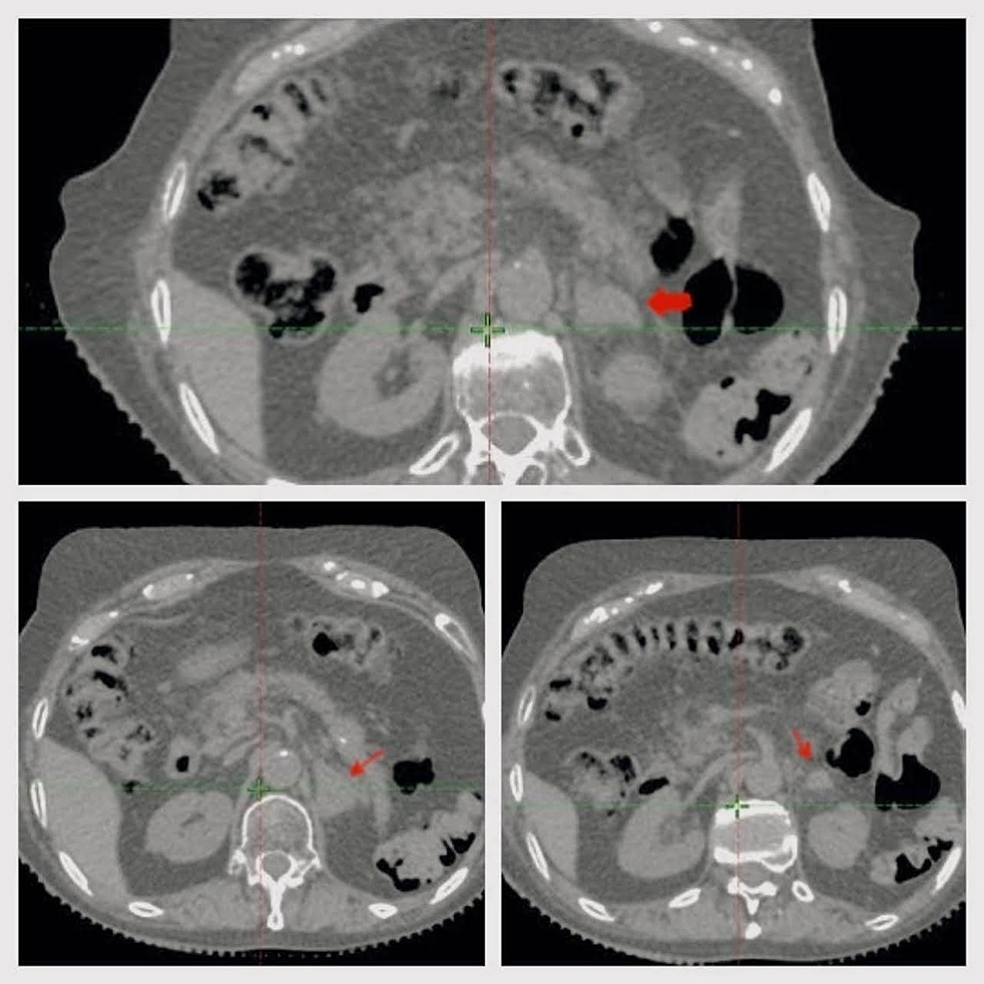
Pre-treatment abdominal CT A mass of soft tissue density can be identified in the left adrenal region, which extends even in front of the upper pole of the left kidney (arrow). No more pathological alterations are identified in the image.

In November 2020, she had another relapse, a mass was discovered on the left adrenal bed, so a positron emission tomography and computed tomography (PET-CT) was performed. The study findings consisted of a hypermetabolic lesion on the left adrenal bed, defined as a unique metastasis, no other hypermetabolic foci were found (Figure 1). With this finding, additional studies were requested, as a body CT, that reported a mass in the left adrenal fossa, measuring 35x25 mm (Figure 2).

**Figure 3:**
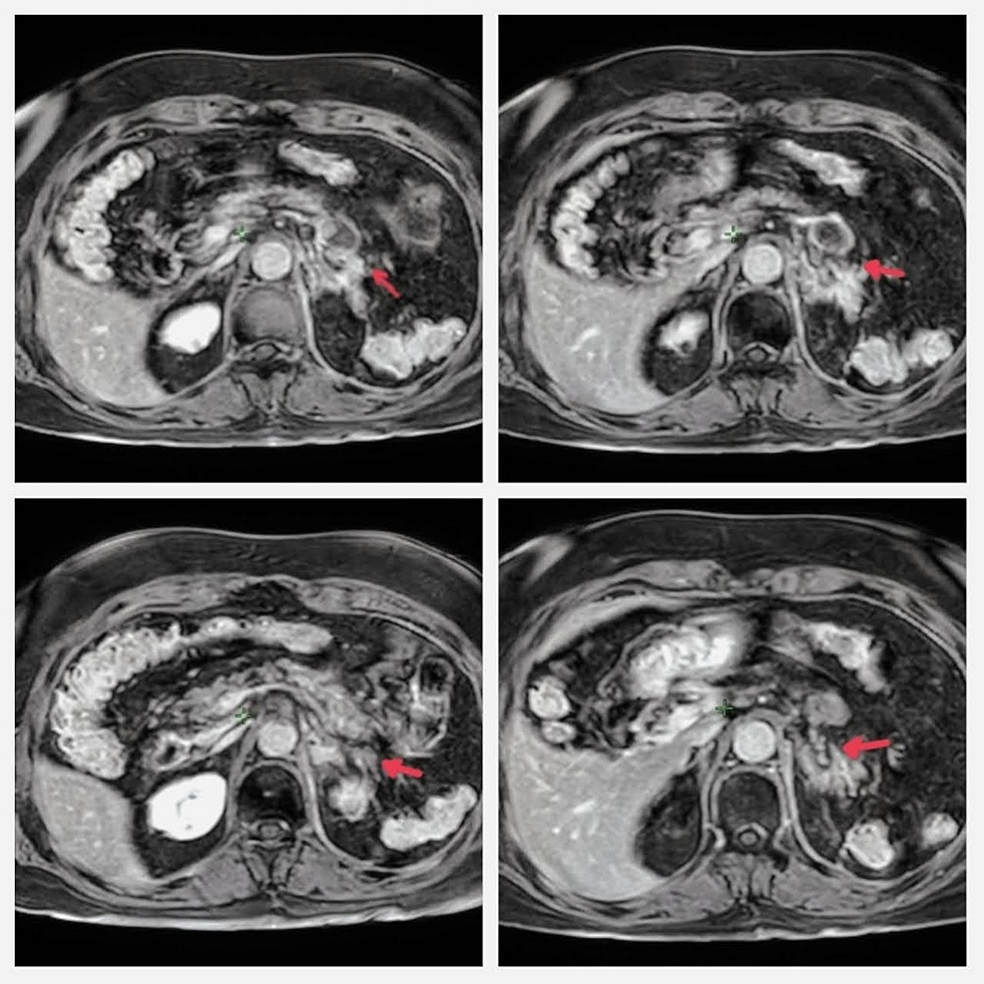
Pre-treatment abdominal MRI In the left suprarenal bed, a hyperintense mass can be identified (arrow) that distorts the hypointensity of the peritoneal fat and extends to the front of the upper pole of the left kidney.

A pelvic and abdominal MRI was also requested in January 2021, reporting a left adrenal fossa heterogenous mass with lobed contours. The measures were 30x25x20 mm, and with the administration of intravenous contrast, it presents a homogeneous enhancement. An aneurismatic dilation was found in the splenic artery, measuring 28x25 mm, which shows a mural thrombus in a half-moon shape inside. There were no other pathological findings in the rest of the study (Figure 3). The total image findings were a left adrenal mass with a possible relation to the previous cervical carcinoma, a sclerosing mesenteritis, a thrombosed aneurysm of the splenic artery (supposably because of the splenectomy), and degenerative changes of the lumbar spine. The patient underwent a biopsy of the mass, and the report confirmed the relationship between the mass and the cancer history, with a diagnosis of metastases of genital origin. With a case analysis, it was determined that surgical management was not possible.

On March 1, 2021, the patient arrives at the radiotherapy department of our hospital, looking for a second opinion. She presented symptoms like pain in her left knee and edema in her lower limbs, accentuated in the afternoons. The physical examination showed a Karnofsky Performance Status (KPS) of 90%, she was conscient, orientated, and lucid; additionally, her abdomen was soft, depressible, not painful, with preserved hydro aerial noises. When examined, in the genital region, the vaginal stump looked normal, and in the limb region, bilateral edema was evidenced. Considering these findings and her previous imaging examinations, it was determined feasible to treat the mass with SBRT. It was decided to supply 40 Gy in five fractions by a TrueBeam LINAC (Palo Alto, CA: Varian Medical Systems, Inc.).

**Figure 4:**
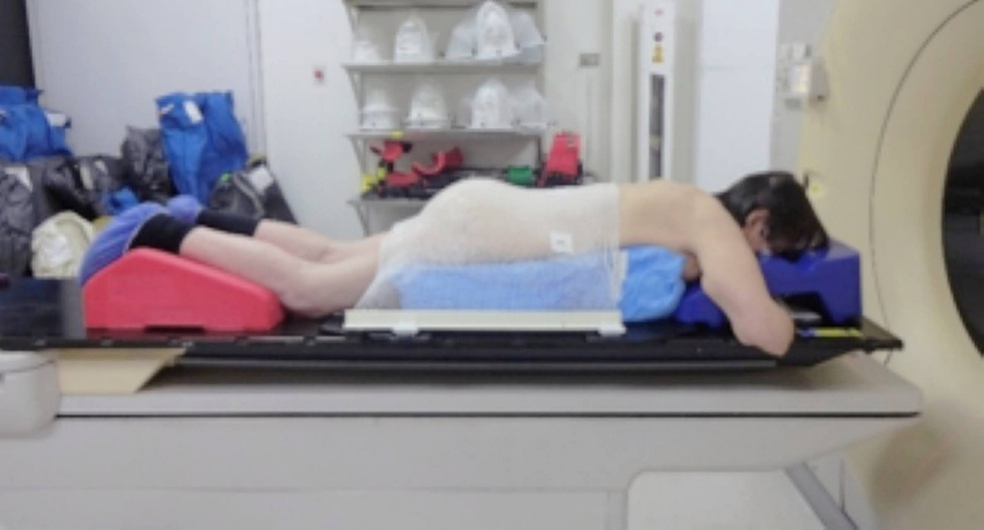
Patient immobilization during the simulation process The image shows the patient in prone position with the head and arms located in the bellyboard-type immobilizer, the thorax and abdomen on the vacuum bag, and back supported by the thermoplastic net.

The patient was placed in a prone position with bellyboard support. The vacuum pouch system with thermoplastic netting was used to immobilize her (Figure 4). The treatment volume was defined based on the simulation obtained images and the PET-CT. This way, the gross tumor volume (GTV) was defined on the hypermetabolic area that was found in PET-CT (Figure 1).

**Figure 5:**
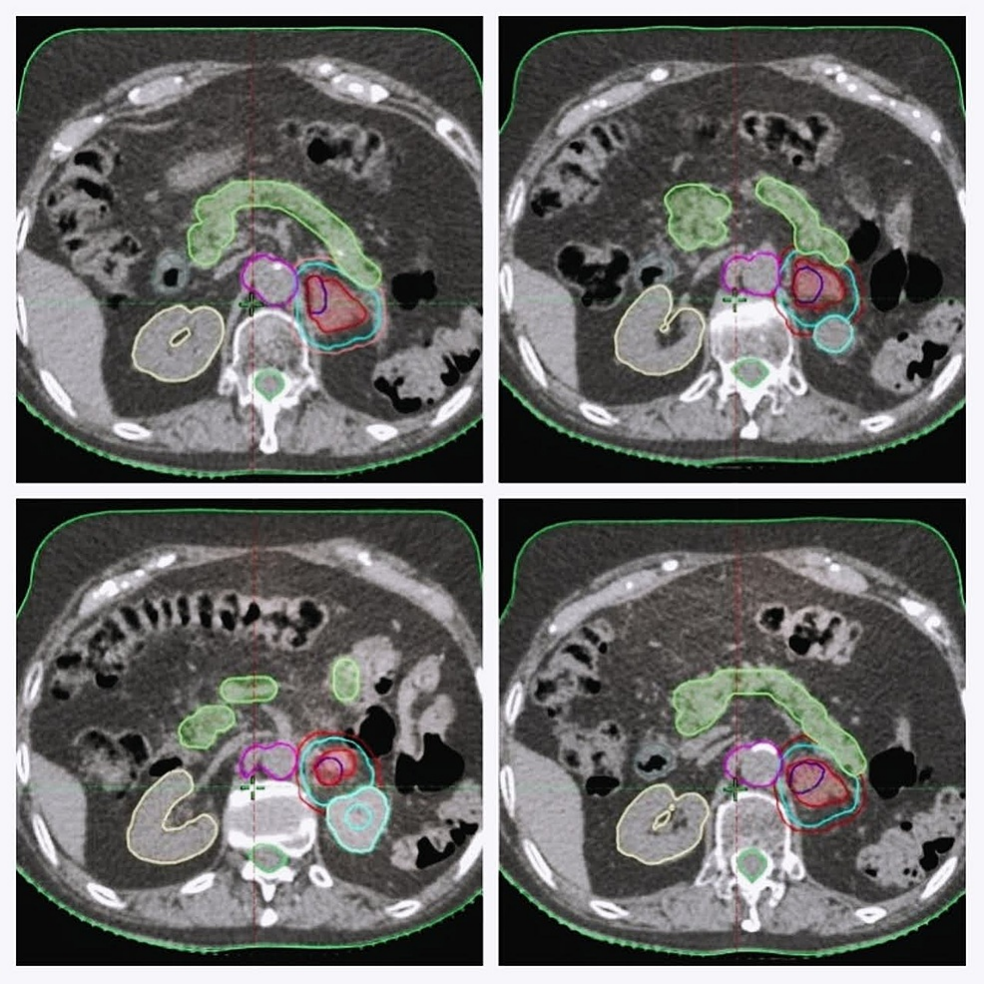
Stereotactic ablative body radiation therapy (SABRT) treatment planning The images show volume definition of GTV in blue, CTV in internal red, ITV in light blue, and PTV in external red. For the definition of GTV, the hypercaptant area established in the PET was considered; for CTV, the mass identified in the diagnostic tomography was considered, even if it did not have significant uptake in the PET; and an ITV margin was assigned considering the extrapolated movement of the patient's breathing to finally assign a short additional margin of PTV. GTV: gross tumor volume; CTV: clinical target volume; ITV: internal target volume; PTV: planning target volume; PET: positron emission tomography

**Figure 6:**
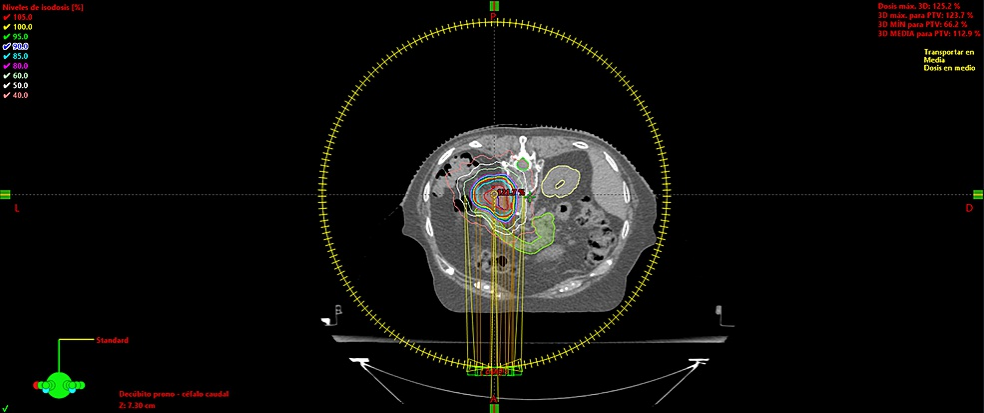
Dosimetric planning The image shows dosimetry of the planned treatment in VMAT. It is important to note how the 50% isodose curve (white color) falls in the vicinity of the PTV. Also, hot spots remained between GTV and CTV. VMAT: volumetric modulated arc therapy; PTV: planning target volume; GTV: gross tumor volume; CTV: clinical target volume

The CTV was decided to contour the entire mass identified on diagnostic tomography, considering the absence of an adrenal gland with its capsule, and the surgical previous manipulation and ITV with two additional millimeters because of the patient breathing movements. The determined planning target volume had an additional 2 mm, excluding the volume of the kidneys, the bowels, and the renal vessels (Figure 5). Planning was based on RapidArc radiotherapy technology (Crawley, United Kingdom: Varian Medical System) (Figure 6). The suitable doses and constraints for risk organs were assumed to be the same as those used for the treatment of metastases on the adrenal gland.

**Figure 7:**
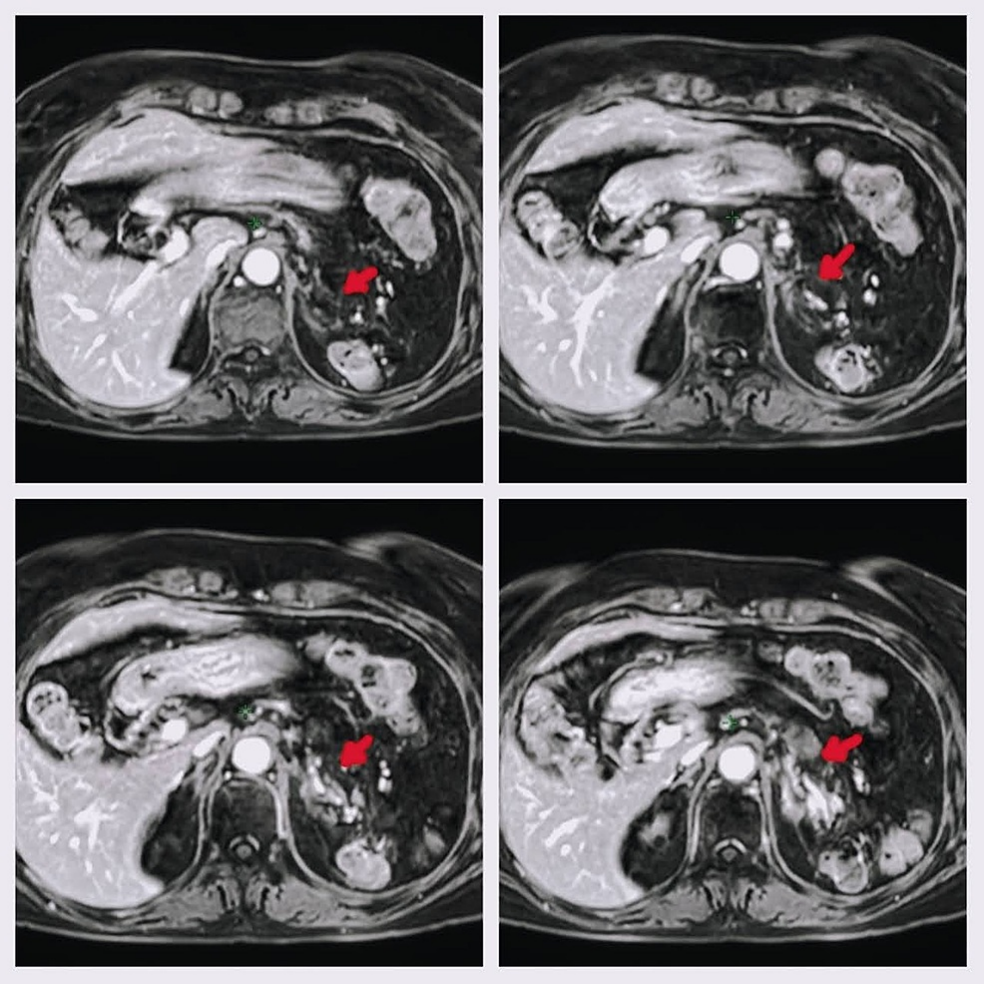
Post-treatment evaluation abdominal MRI The images show hypointense bed (arrow) that has remained after the treatment in the areas where they were previously occupied by the hyperintense mass. Hyperintense remnants can also be identified, especially near the vertebral body. However, a significant decrease in the size of metastasis can be identified.

Treatment was delivered on March 26, 27, and 29-31, 2021, using the tomography verification system, cone-beam CT, before each session, with no setbacks. Urine samples were taken during the treatment and 15 days after the last session for the quantification of blood in urine, with normal findings. Due to the pandemic, the patient prorogated the date of her next medical check-up by six months, until September 2021, when she had an MRI. The imaging study showed a mass in the left adrenal bed, measuring 16x18 mm, with a poorly defined contour, a homogenous signal, and a predominantly peripheral enhancement, with a 50% reduction compared with the study previously performed in January of that same year (2021) (Figure 7). By January 2022, the patient went for a follow-up appointment showing no symptomatology and with an excellent functional status.

## Discussion

This case consists of a patient with a primary uterine cervical carcinoma that evolved to an oligometastatic case, with tumoral invasion of the left adrenal bed. A primary cervical carcinoma and a secondary adrenal tumor both are usual situations, however, the cases where they occur together are rare [2]. Cervical carcinoma is one of the most common malignant tumors in women, but it usually spread secondarily to the lymphatic system. The most common sites of distant spread are lungs, bones, liver, and brain [3].

The adrenal glands have favorable anatomy to be a usual metastatic site, as they have an abundant sinusoidal blood supply [19]. They are, in fact, the fourth most common metastatic site, with a metastasis incidence of 9-27% [19,20]. The most common primary tumors that spread to the adrenal glands are from the lungs, breasts, gastrointestinal system, and skin (melanomas) [20]. As mentioned earlier, adrenal metastasis from a primary cervix carcinoma is rare, and the case reports found in the literature are scarce; there is a cohort study with 34 patients that shows, however, an incidence of 6% of cervix carcinoma spreading to an adrenal metastasis [7]. In addition to adrenal metastasis being unlikely in cervix carcinoma, our patient had previous adrenalectomy, and the metastasis was in the adrenal bed [20]. We could not find any studies with a similar clinical situation, except for a case were a uterine papillary serous carcinoma originated an adrenal secondary tumor, and after the adrenalectomy, there was PET scan evidence of metabolic uptake in the adrenal bed, but it was because of the patient denied chemotherapy [20].

The first-line management of adrenal metastasis is a surgical gland resection, mainly by a laparoscopic approach [6]. A surgical approach has better outcomes than non-invasive treatments, with a median overall survival (OS) almost twice as long for patients who underwent surgery compared with patients receiving other treatments [19]. Another case report with an adrenal secondary tumor originating from an endometrial carcinoma showed a good response to laparoscopic surgery [8]. This therapeutic approach can not apply to our patient who had adrenalectomy previous to the metastases, and another therapy had to be chosen. Non-invasive therapies are usually considered palliative measures for secondary adrenal tumors, as the prognosis for those lesions is poor [20]. In a case report of adrenal metastasis from an endometrial tumor, the patient received chemotherapy as a palliative measure, and the patient died three months after the metastasis diagnosis [8]. SBRT has been proven to be a good palliative measure for adrenal metastasis, as it helps to relieve pain in 85.7% of patients [12]. A case series shows seven patients in whom SBRT was used only to relieve the pain caused by the tumors, and most patients end up deceased [13]. However, it has been shown that SBRT can increase the life expectancy in patients with oligometastatic tumors involving the adrenal gland, compared with patients receiving lower palliative radiation doses [16,20].

The dosage decision for our patient was based on evidence of adrenal metastasis treatment from different primary tumor origins because of the lack of similar cases to ours. Evidence shows that a higher BED is associated with a higher LC, with values of 72 or higher [12,20]. The most recommended doses range from 30 to 60 Gy in three to 10 fractions, which is why we used 40 Gy in five fractions on our patient [18,20]. Additionally, it was proven that SBRT with RapidArc should be considered the first radiation treatment option for its simplicity, so we planned the radiation therapy with RapidArc based on that recommendation [20]. Most studies reported limited toxicity with SBRT, with a grade lower than three in all cases [16].

The five-year survival rate for localized uterine cervical cancer is 91.5%, meanwhile, it drops to 16.5% when it is metastatic [3]. As mentioned before, the prognosis for adrenal metastasis is poor, so the overall survival for our patient is not expected to be long [20]. Concerning the LC of patients with adrenal metastasis after SBRT, in a retrospective cohort study, the mean value was 14.6±1.8 months, with a two-year LC of 40.7±15.8% [14]. The median OS reported in the literature varies from 22-26 months, with a two-year OS of 87.6±6.1% [14,17,20]. The treatment of our patient was ended on March 31, 2021, and in her last control in January 2022, there was a 50% reduction of the tumor, and no evidence of metastasis in other sites. To this date, the LC and OS of our patient are 14 months, which are consistent with most of the outcomes of SBRT in adrenal metastasis despite the particular characteristics, as it is a mass in the adrenal bed with the loss of the integrity of the capsule that surrounds an intact gland and the surgical manipulation of said area.

## Conclusions

The current study appears to be the first case reported in the literature of a uterine cervical cancer metastasis localized in the adrenal bed in a previously adrenectomized patient. It serves as a precedent for the use of stereotactic ablative body radiation therapy (SABRT) with special considerations of GTV, CTV, and ITV for the treatment of this kind of tumor. The considerations of CTV and ITV could be extrapolated to the ablative management of other masses located in the retroperitoneal region. Our patient continues to be asymptomatic 14 months after diagnosis, which is consistent with most of the outcomes of SBRT in adrenal metastasis despite the surgical manipulation of the area and the special considerations in this case.
